# Hyperbaric oxygen preconditioning ameliorates blood-brain barrier damage induced by hypoxia through modulation of tight junction proteins in an *in vitro* model

**DOI:** 10.3325/cmj.2016.57.51

**Published:** 2016-02

**Authors:** Lei Hao, Xiuming Guo, Can Zou, Huchuan Zhou, Hong Tian, Yubo Zhang, Chuan Song, Lei Liu

**Affiliations:** 1 Department of Neurology, No. 324 Hospital of People’s Liberation Army, Chongqing, China; 2 Department of Neurology, The First Affiliated Hospital of Chongqing Medical University, Chongqing, China; The first two authors contributed equally.

## Abstract

**Aim:**

To explore the effects of hyperbaric oxygen preconditioning (HBOP) on the permeability of blood-brain barrier (BBB) and expression of tight junction proteins under hypoxic conditions *in vitro*.

**Methods:**

A BBB *in vitro* model was constructed using the hCMEC/D3 cell line and used when its trans-endothelial electrical resistance (TEER) reached 80-120 Ω · cm^2^ (tested by Millicell-Electrical Resistance System). The cells were randomly divided into the control group cultured under normal conditions, the group cultured under hypoxic conditions (2%O_2_) for 24 h (hypoxia group), and the group first subjected to HBOP for 2 h and then to hypoxia (HBOP group). Occludin and ZO-1 expression were analyzed by immunofluorescence assay.

**Results:**

Normal hCMEC/D3 was spindle-shaped and tightly integrated. TEER was significantly reduced in the hypoxia (*P* = 0.001) and HBOP group (*P* = 0.014) compared to control group, with a greater decrease in the hypoxia group. Occludin membranous expression was significantly decreased in the hypoxia group (*P* = 0.001) compared to the control group, but there was no change in the HBOP group. ZO-1 membranous expression was significantly decreased (*P* = 0.002) and cytoplasmic expression was significantly increased (*P* = 0.001) in the hypoxia group compared to the control group, although overall expression levels did not change. In the HBOP group, there was no significant change in ZO-1 expression compared to the control group.

**Conclusion:**

Hyperbaric oxygen preconditioning protected the integrity of BBB in an *in vitro* model through modulation of occludin and ZO-1 expression under hypoxic conditions.

Ischemic tolerance is an endogenous protective mechanism that refers to the ability of a sublethal stimulus to induce tolerance to a subsequent lethal ischemic injury. It was first demonstrated in neuronal cells of the gerbil hippocampus (1), after which it has aroused a considerable interest as a possible therapeutic modality for ischemic brain diseases. However, to expose patients to brief periods of ischemia is both impractical and unsafe. Chemical preconditioning substances that can induce ischemic tolerance, such as endotoxins, cytokines, metabolic inhibitors, potassium, chloride, and neurotoxin 3-nitro-propionic acid (1-5) have also been investigated but were found to have limited clinical application due to toxicity and side effects.

A variety of experimental models of cerebral ischemia have found that hyperbaric oxygen preconditioning (HBOP) induces ischemic tolerance and attenuates cerebral injury (6-17). Its protective effect is also visible in other conditions leading to oxidative stress, with final anti-apoptotic result, as well as modulation of neutrophin and immune systems (6-17).

The blood-brain barrier (BBB), a highly selective permeability barrier, consists of tight junctions (TJ) between capillary endothelial cells, the basal lamina, pericytes, and astrocyte end-feet (18). It plays an important role in maintaining cerebral homeostasis by restricting molecular movement from the cerebral capillaries to the brain tissue. BBB breakdown can result in a vasogenic edema, hemorrhage, and neuronal cell death, all of which can contribute to the pathophysiology of cerebral ischemic diseases (19). TJs between cerebral endothelial cells are formed by complex interactions of cytoskeletal proteins and tight junction proteins (TJPs), including claudins, occludin, zonula occludens (ZO), and cingulin (20). TJPs increase endothelial electrical resistance and decrease paracellular permeability (21). Changes in their expression can lead to the loss of BBB integrity and BBB breakdown (22).

HBOP has been associated with reduced brain edema, decreased infarct volume, and improved neurological function (6-17), but it is not clear whether it directly affects the BBB, particularly TJPs expression. This should be clarified in order to find new therapeutic strategies to attenuate BBB permeability in cerebral ischemic disorders. Therefore, the aim of this study was to examine the HBOP effect on hypoxia-induced BBB breakdown *in vitro* and the changes of occludin and ZO-1 expression.

## Materials and methods

### Materials

Ascorbic acid was purchased from Sigma (St Louis, MO, USA); chemically defined lipid concentrate from Invitrogen (Carlsbad, CA, USA); endothelial growth basal medium-2 (EBM-2) from Lonza (Walkersville, MD, USA); human basic fibroblast growth factor (bFGF) from Cell Signaling Technology (Danvers, MA, USA); hydrocortisone from Fisher Scientific (Pittsburg, PA, USA); fetal bovine serum (FBS) from Hyclone (Logan, UT, USA); penicillin/streptomycin from Cellgro Mediatech, Inc. (Manassas, VA, USA); type I collagen from R&D System (Minneapolis, MN, USA); TritonX-100 and bovine serum albumin (BSA) both from Sigma-Aldrich (St Louis, MO, USA); primary antibodies for occludin and ZO-1 (both diluted 1:100; Abcam Cambridge, MA, USA); fluorophore-conjugated secondary antibody from Proteintech Group (diluted 1:200; Chicago, IL, USA); and 4′,6-diamidino-2-phenylindole from Sigma-Aldrich.

### Experimental design

*In vitro* BBB cultures were given random numbers using Excel software and were divided into three groups: control group; hypoxia group, cultured in an anaerobic chamber (Thermo Forma Scientific, Hudson, NH, USA) filled with an anoxic gas mixture (2% O2, 5% CO2, and 93% N2) at 37°C for 24 h; and HBOP group, subjected to hyperbaric oxygen conditioning for 2 hours before culturing in an anaerobic chamber. Trans-endothelial electrical resistance (TEER) was measured using Millicell-Electrical Resistance System (ERS, Millipore, Billerica, MA, USA). Occludin and ZO-1 expression was analyzed using immunofluorescence assay.

### In vitro BBB model

The hCMEC/D3 cell line (EMD Millipore, Temecula, CA, USA, catalog number SCC066) is derived from microvascular cells of human brain tissue and is a reliable *in vitro* model for understanding molecular and cellular regulation of human BBB integrity (23-25). The cells were cultured in EBM-2 with 5% FBS supplemented with penicillin/streptomycin, hydrocortisone (1.4 μM), ascorbic acid (5.0 μg/mL), chemically defined lipid concentrate (1.0%), 4-(2-hydroxyethyl)-1-piperazineethanesulfonic acid (HEPES) (10.0 mM), and bFGF (1.0 ng/mL), and maintained at 37°C, 5% CO_2_, and 95% relative humidity. They were seeded at a density of 2 × 10^5^ cells/well onto Transwell inserts (0.4 μm pore size, 24 mm diameter; Corning, NY, USA), coated with type I collagen. The cell culture medium was changed every 3 days until the cell monolayer became confluent. The formation of cell monolayer and verification of TJs was evaluated by measuring TEER values, which ranged from 80-120 Ω · cm^2^.

### Hyperbaric oxygen preconditioning (HBOP)

HBOP was performed in a temperature and humidity controlled hyperbaric incubator (OxyCure 3000, OxyHeal® Health Group, National City, CA, USA). The pressure duration was 280 kPa-60 min, which is frequently used in animal and cell studies (26). The compression and decompression were both carried out n 5 min. The chamber was flushed and compressed with pure 100% oxygen. All the pressures described are absolute pressures.

### TEER measurement

TEER, a key BBB characteristic, has been extensively used to measure TJ function resistance of the endothelial cells in BBB Transwell models using an epithelial voltohmmeter (27,28). TEER was measured when the cells formed a confluent monolayer using Millicell-ERS equipment at 37°C and a heating plate to avoid temperature fluctuation. Background electrical resistance including filter and medium was subtracted from each reading. TEER values were calculated as Ω · cm^2^ by multiplying the surface area of the Transwell insert.

### Immunofluorescence assays

Expression and distribution of occludin and ZO-1 in hCMEC/D3 cells was analyzed by immunofluorescence assay. The cells from three groups were fixed with 4% paraformaldehyde for 20 min and permeabilized with 0.1% TritonX-100 for 20 min. They were blocked in 5% BSA for 2 h at room temperature and incubated at 4°C overnight with appropriate primary antibodies for occludin and ZO-1. The cells were rinsed three times with PBS and incubated with fluorophore-conjugated secondary antibody for 2 h at room temperature in darkness. The nucleus was stained with DAPI for 1 min. The samples were observed and photographed with an immunofluorescence microscope (BX51, Olympus, Tokyo, Japan). Finally, the images were quantified using the image Pro Plus Version 6.0 software (Media Cybernetics, Inc., Rockville, MD, USA).

### Statistical analysis

Data are presented as mean ± standard deviation. Multiple comparison of group means was performed by Tukey honestly significant difference test with Tamhane T2 used for post hoc comparison. Statistical analysis was performed using SPSS 13.0 (SPSS Inc., Chicago, IL, USA). *P*-values lower than 0.05 were considered significant.

## Results

### BBB model establishment

**Figure 1:**
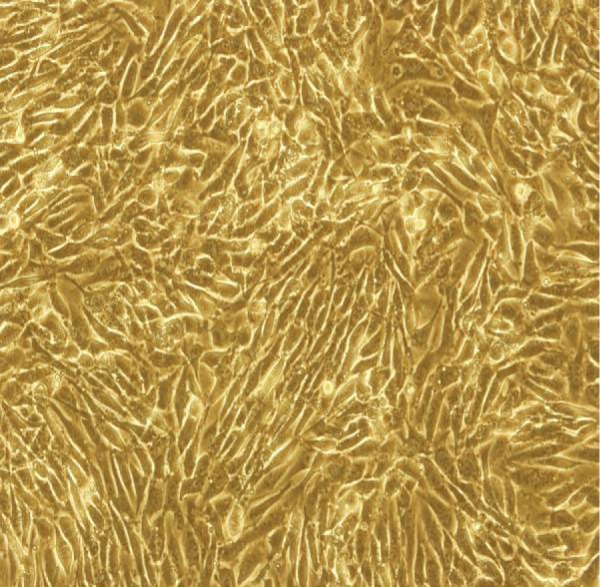
Phase contrast microscopy of hCMEC/D3 (200 × ). The cells were cultured in endothelial growth medium-2 and attained confluence after 3-5 days.

The hCMEC/D3 cells attained confluence after 3-5 days. They showed elongated, tightly packed, contact inhibited morphology (Figure 1), with a TEER value of 107.17 ± 10.41 Ω · cm^2^. This indicated that BBB model *in vitro* was successfully established and suitable for use.

### Effect of HBOP on TEER of BBB in vitro under hypoxic conditions

**Figure 2:**
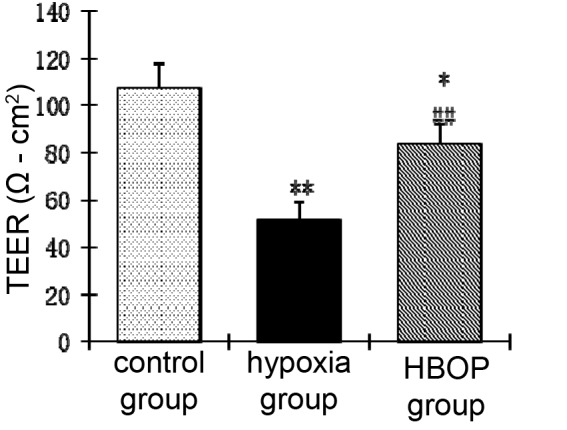
Trans-endothelial electrical resistance (TEER) value in different groups. *vs control group, *P* = 0.014; **vs control group *P* ≤ 0.001; ^##^vs hypoxia group, *P* = 0.001. HBOP – hyperbaric oxygen preconditioning.

TEER value significantly decreased in the hypoxia group compared to the control group (50.02 ± 6.87 Ω · cm^2^ vs 107.17 ± 10.41 Ω · cm^2^, *P* ≤ 0.001). In HBOP group, it significantly increased (83.81 ± 8.22 Ω · cm^2^) compared to the hypoxia group (*P* = 0.001), but was still significantly lower than in the control group (*P* = 0.014) (Figure 2). These results indicate that HBOP can prevent the effects of hypoxia on BBB permeability.

### Effect of HBOP on TJPs in vitro under hypoxic conditions

In the control group, occludin showed continuous membranous expression and very low cytoplasmic expression. In the hypoxia group, continuous membranous expression was significantly decreased compared to the control group, and in the HBOP group it was significantly increased compared to the hypoxia group. Relative mean optical density of occludin was significantly lower in the hypoxia group than in the control group (0.80 ± 0.10 vs 1.38 ± 0.11, *P* = 0.001), and in the HBOP group it was significantly higher than in the hypoxia group (1.32 ± 0.08, *P* = 0.008). There was no significant difference between the HBOP and control group.

**Figure 3:**
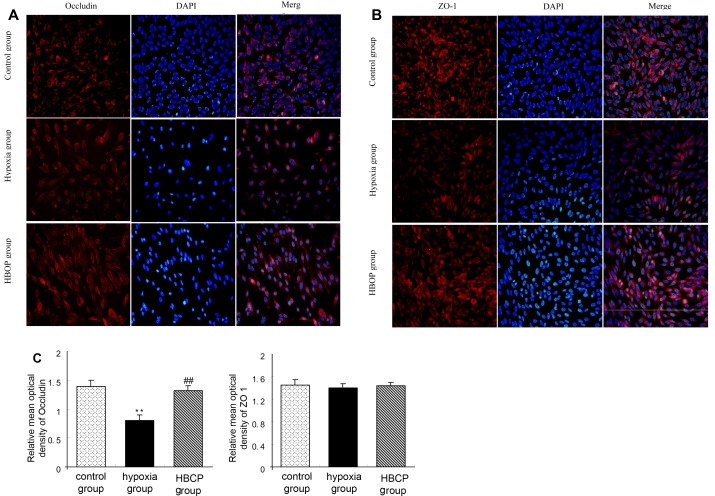
The effect of hyperbaric oxygen preconditioning (HBOP) on tight junction proteins (TJP) in the blood-brain barrier (BBB) *in vitro* under hypoxic condition. TJPs expression was tested by immunofluorescence in the control, hypoxia, and HBOP group. Distribution and expression of occludin (**A**) and zonula occludens protein (ZO-1) (**B**). Occludin (red) and ZO-1 (red) were labeled with secondary antibody against anti-occludin antibody and against anti-ZO-1 antibody. The nucleus (blue) was labeled with 4′,6-diamidino-2-phenylindole (DAPI). Images represent three independent experiments. (**C**) Relative mean optical density of occludin and ZO-1 immunofluorescence. Data are presented as mean ± standard deviation (n = 3, each group). **P = 0.001 vs control group; ^##^*P* = 0.008 vs hypoxia group.

In the control group, ZO-1 mainly showed membranous expression and very low cytoplasmic expression, which gradually increased in the hypoxia group. Interestingly, there was no significant difference in the expression and distribution of ZO-1 between the HBOP (1.43 ± 0.06) and control group (1.45 ± 0.10), demonstrated by immunofluorescence (Figure 3). Relative mean optical density of ZO-1 in the hypoxia group (1.40 ± 0.07) was comparable to the control group (1.44 ± 0.10), indicating there was no change in the overall ZO-1 expression levels. However, ZO-1 membranous expression was significantly decreased (0.32 ± 0.04, *P* = 0.002) and cytoplasmic expression was increased (1.15 ± 0.13, *P* = 0.001) in the hypoxia compared to the control group (1.42 ± 0.08, 0.10 ± 0.03).

## Discussion

The present study found that HBOP ameliorated the effect of hypoxic damage on the BBB, which was probably associated with TJPs expression. HBOP has been shown to reduce BBB permeability in several animal models of ischemia (6,12-16). In the present study, TEER value decreased after hypoxia, but the decrease was mitigated by HBOP before hypoxia induction. These results suggested that HBOP can protect the BBB from breakdown after hypoxia, consistent with results in animal models (6-17).

Changes in TJPs expression and distribution are closely associated with changes in BBB permeability (18). Opening of TJS is regulated by complicated TJPs, such as transmembrane proteins, members of the peripheral membrane protein family, and adhesion molecules (29), while occludin is responsible for their sealing. Disruption of occludin expression alone is enough to cause functional changes in TJs (30,31). ZO-1 serves as a bridge between transmembrane proteins and skeleton proteins, which is important for the stability and function of TJPs (32,33). In addition, the loss of the permeability barrier function in the early phase of hypoxia-ischemia involves endothelial TJ dysfunction, which is associated with relocation and up-regulation of occludin and ZO-1 (34,35). Previous research yielded conflicting results on the effects of hypoxia on TJPs expression. Several studies showed that hypoxia decreased occludin and ZO-1 expression in cultured endothelial cells (36-39). However, other studies observed no significant changes in protein expression of occludin and ZO-1 in bovine bone marrow endothelial cells under hypoxia condition (35,40) or at 6 h exposure to hypoxia (41). Some researchers found reduced occludin expression but unchanged ZO-1 expression in an *in vivo* model of prolonged tissue hypoxia (42). TJPs expression may be affected by several factors, including the experimental setting (*in vitro* vs *in vivo*), culturing conditions, exposure time, cell type, or test methods. In the present study, occludin and ZO-1 exhibited continuous membranous expression under normal conditions and a discontinuous expression after hypoxia. Notably, cytoplasmic expression of ZO-1 increased after hypoxia, but that of occludin did not change. Mean optical density measurement showed that hypoxia decreased occludin expression, but did not affect the overall ZO-1 expression. In other words, ZO-1 expression was transferred from the membrane to the cytoplasm. HBOP before hypoxia reversed the decrease in occludin expression and prevented ZO-1 relocation. We believe that this might explain TEER increase in the HBOP group compared to hypoxia group. These results suggest the potential of HBOP to protect the BBB integrity, but they still have to be validated *in vivo*.

In conclusion, our study demonstrates that HBOP can protect the integrity of BBB *in vitro* model compromised by hypoxia by modulating occludin and ZO-1 expression. Further studies are needed to investigate the effect of HBOP on other TJPs and elucidate specific molecular mechanisms involved in these processes.
